# Interaction between Electromechanical Fields and Carriers in a Multilayered Piezoelectric Semiconductor Beam

**DOI:** 10.3390/mi13060857

**Published:** 2022-05-30

**Authors:** Renzhong Hong, Wanli Yang, Yunbo Wang

**Affiliations:** 1 Department of Mechanics, School of Aerospace Engineering, Hubei Key Laboratory of Engineering Structural Analysis and Safety Assessment, Huazhong University of Science and Technology, Wuhan 430074, China; hrz_hust20@163.com; 2 Department of Microelectronics, School of Optical and Electronic Information, Huazhong University of Science and Technology, Wuhan 430074, China

**Keywords:** piezoelectric semiconductor, carriers, polarized charges, potential-barrier/well, tuning

## Abstract

This study discusses the interaction between electromechanical fields and carriers in a multilayered ZnO beam where the c-axis of every two adjacent layers is alternately opposite along the thickness direction. A multi-field coupling model is proposed from the Timoshenko beam theory together with the phenomenological theory of piezoelectric semiconductors, including Gauss’s law and the continuity equation of currents. The analytical solutions are obtained for a bent beam with different numbers of layers. Numerical results show that polarized charges occur at the interfaces between every two adjacent layers due to the opposite electromechanical coupling effects. It was found that a series of alternating potential-barrier/well structures are induced by the polarized charges, which can be used to forbid the passing of low-energy mobile charges. Moreover, it was also observed that the induced polarized charges could weaken the shielding effect of carrier redistribution. These results are useful for the design of piezotronic devices.

## 1. Introduction

Piezoelectric material is a kind of material with a mechanical–electric coupling effect. When it is deformed by stress, electric polarization will occur inside it. This phenomenon is called the piezoelectric effect. Piezoelectric materials can create an electric field due to deformation or create deformation due to an electric field. The inherent mechanical–electrical coupling effect makes piezoelectric materials widely used for applications such as energy harvesters, sensors, memories, and batteries [1,2,3,4]. Piezoelectric materials can be dielectrics and semiconductors. Piezoelectric semiconductor materials possess both piezoelectricity and semiconducting properties, and they are frequently utilized in electronic devices. They are differentiated from pure piezoelectric materials by the existence of the free charges, which further enrich the process of the electromechanical conversion. As is known, the piezoelectric polarization electric field induced by deformation drives carriers to redistribute, while the redistribution of carriers produces a shielding effect on the electric field itself in turn. Therefore, on the basis of the interaction of carriers and electromechanical fields, electronic devices not only can be tunable by the classical electrical form, but also by the mechanical form. This amazing feature has formed new research fields called piezotronics and piezo-phototronics [5], which include many strain tunable devices, i.e., piezoelectric field-effect transistors [6,7,8], transducers [9,10], laser detectors [11], solar cells [12,13], flexible data storages [14], and so on.

However, previous research has been mostly focused on the single structure of fibers, tubes, belts, and spirals [15,16,17,18,19]. As we know, composite laminated structures are widely utilized in the aerospace, naval, civil, and mining industries due to the advantage of the designability of each layer. Thus, significant attention has been paid to this topic recently. Sharma et al. [20] studied the propagation of interfacial surface waves in a two-layered composite structure consisting of a transversely isotropic piezoelectric substrate and an isotropic non-piezoelectric semiconductor layer. Othmani et al. [21,22] used the Legendre polynomial method to investigate Rayleigh–Lamb and guided Lamb waves propagating in the multilayered semiconductor structures. However, in their study, the space charges of the semiconductor were neglected. Luo et al. [23] studied the bending of a composite fiber of piezoelectric dielectrics and nonpiezoelectric semiconductors. Cheng et al. [24] further studied the extension of a composite fiber of piezoelectric dielectrics and nonpiezoelectric semiconductors. The two studies above found that the composite fiber exhibits piezotronic couplings like a homogeneous piezoelectric semiconducting fiber [23,24]. Jiao et al. [25] analyzed the wave propagation through a piezoelectric semiconductor slab sandwiched by two piezoelectric half-spaces. Recently, Tian et al. [26,27] studied wave propagation properties of layered piezoelectric semiconductor plates with imperfect and perfect interfaces. Liang et al. [28] investigated the electrical behaviors of a PN junction in a composite fiber made of piezomagnetic and piezoelectric semiconductor layers under a constant magnetic field. Qu et al. [29] investigated the interaction between mechanical fields and the motion of charge carriers in a composite beam of flexoelectric (nonpiezoelectric) and semiconductor layers. This study provides a new means for constructing electromechanical semiconductor devices and extends piezotronic devices.

Although significant research have been carried out on layered piezoelectric semiconductor structures, most of these have been homogenous structures. It is noted that hetero-structures have non-substitutable superiority in interface engineering compared with homo-structures. Thus, the related study of hetero-structures is a hot topic. As we know, a graphene bilayer can be treated as a simple heterojunction with Van der Waals forces acting between the layers, which creates some amazing properties different from monolayer structures [30,31]. That is to say, the homo-structure can serve as a special hetero-structure (quasi-hetero-structure) after some preprocessing. For example, some properties of a hetero-junction appear in a piezoelectric homo-junction with different *c*-axis directions [32,33]. The present study is motivated by the above works to investigate the electrical responses of a multilayered ZnO beam composed of opposite polarization directions along the length-direction under a transverse end force. In this paper, we use the conventional phenomenological theory composed of the equations of linear piezoelectricity and the equations of the conservations of charge of electrons to describe the basic behaviors of a cantilever ZnO beam. The one-dimensional theory for the bending of a multilayered ZnO beam with shear deformation is derived based on the Timoshenko beam theory. Then, the mechanical tuning laws of the electrostatics are carefully studied in Section 4. Finally, some interesting conclusions are drawn in Section 5.

## 2. One-Dimensional Equations for a Multilayered Piezoelectric Semiconductor Beam

The piezoelectric coefficient $e_{33}$ of ZnO is relatively large compared with other wurtzites, such as GaN and CdS. Thus, we chose it as the material of the structure being researched.

**Figure 1:**
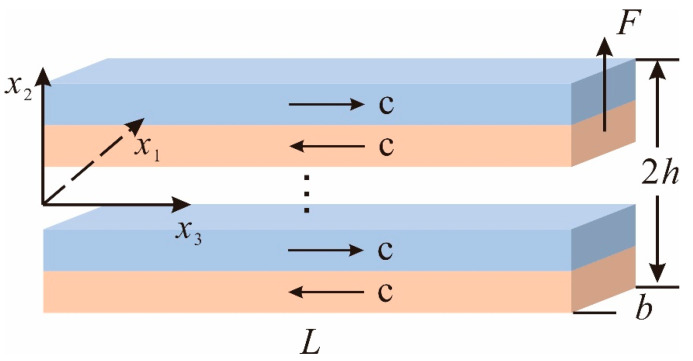
A multilayered ZnO beam with alternating opposite polarization directions along the thickness direction.

As shown in Figure 1, a multilayered ZnO beam is considered with alternating opposite polarization directions along the thickness direction. It should be noted that this multilayered beam can be prepared by MOCVD [34]. The left end of the cantilever is fixed, and the right end is under the action of a transverse shear force *F*. The positive direction of the *c*-axis of ZnO is defined along the positive $x_{3}$ direction. The length and thickness of the beam are denoted as L and 2h, respectively. In the following, k means the number of layers. We choose the geometrical middle plane of the beam as the coordinate plane. $x_{1}$, $x_{2}$, and $x_{3}$ represent the width, thickness, and axial directions, respectively.

We are mainly interested in the electrical responses along the thickness direction, because the axial electric field is almost zero except at the two ends [35]. Therefore, we have

(1) $$\varphi \left(x,t\right)≅\varphi \left(x_{2},t\right)$$

Based on the Timoshenko beam theory, we make the following approximations of the relevant mechanical displacements and carrier concentrations [36,37,38,39]:

(2) $$u_{2}\left(x_{3},t\right)≅v\left(x_{3},t\right)$$

(3) $$u_{3}\left(x,t\right)≅w\left(x_{3},t\right)+x_{2}\psi \left(x_{3},t\right)$$

(4) $$\Delta n\left(x_{2},t\right)≅\Delta n\left(x_{2},t\right)$$

where v, w and ψ are the flexural displacement, extensional displacement, and shear deformation, respectively. The relevant strains and electric fields can be written into

(5) $$S_{3}=u_{3,3}=w_{,3}+x_{2}\psi_{,3},\;S_{4}=2S_{23}=u_{2,3}+u_{3,2}=v_{,3}+\psi ,$$

(6) $$E_{2}=-\varphi_{,2},\;E_{3}=-\varphi_{,3}=0.$$

For bending in the $x_{2}\text{-}x_{3}$ plane, the main stress components are $T_{3}$ and $T_{4}$. Therefore, we introduce the following stress relaxation [40]:

(7) $$T_{1}=T_{2}=T_{5}=T_{6}≅0.$$

In addition, the null current condition in the static bending requires [41,42,43]

(8) $$J_{2}^{n}=qn\mu_{11}^{n}E_{2}+qD_{11}^{n}n_{,2}=0,\;J_{3}^{n}=qn\mu_{33}^{n}E_{3}+qD_{33}^{n}n_{,3}=0.$$

In the above, n represents carrier concentration. $\mu_{11}^{n}$ and $\mu_{33}^{n}$ are the mobility of electrons; $D_{11}^{n}$ and $D_{33}^{n}$ are the diffusion coefficients. It is noted that a tiny fluctuation of carrier concentration will be induced in a small shear force *F*, such that the first *n* in Equation (8) can be approximately written into $n_{0}$. Moreover, $E_{3}=0$ implies that $n_{,3}=0$, which further means the redistribution of the carriers is only limited in the cross-section. Thus, the carrier concentration gradients can be written as

(9) $$\Delta n_{,2}≅\Delta n_{,2}\left(x_{2},t\right),\;\Delta n_{,3}=0.$$

In the following, we use the superscript symbol “(*i*)” to indicate the related quantity of the $i^{th}$ ZnO layer. The piezoelectric coefficients ($e^{\left(i\right)}$) of adjacent two layers are opposite due to the opposite polarization direction. As is well known, piezoelectric coefficients are an odd-order tensor. Therefore, Equation (10) exists for two adjacent layers with opposite c-axis directions:

(10) $$e^{\left(i\right)}+e^{\left(i+1\right)}=0,i=1,2\ldots k-1$$

while the elastic and dielectric coefficients ($c^{\left(i\right)}$ and $\varepsilon^{\left(i\right)}$) are still the same due to their even-order features, that is

(11) $$c^{\left(1\right)}=c^{\left(2\right)}=\ldots =c^{\left(k\right)}=c,\;\varepsilon^{\left(1\right)}=\varepsilon^{\left(2\right)}=\ldots =\varepsilon^{\left(k\right)}=\varepsilon$$

By using the following compact notation: 11→1, 22→2, 33→3, 23→4, 31→5, and 12→6, the constitutive relations for the *i*-th layer can be written as [40]

(12) $$T_{3}={\bar{c}}_{33}S_{3},\;T_{4}=c_{44}S_{4}-e_{15}^{\left(i\right)}E_{2},$$

(13) $$D_{3}={\bar{e}}_{33}^{\left(i\right)}S_{3},\;D_{2}=e_{15}^{\left(i\right)}S_{4}+\varepsilon_{11}E_{2},$$

where

(14) $${\bar{c}}_{33}=c_{33}-2{\left(c_{13}\right)}^{2}/\left(c_{11}+c_{12}\right),\;{\bar{e}}_{33}^{\left(i\right)}=e_{33}^{\left(i\right)}-2e_{31}^{\left(i\right)}c_{13}/\left(c_{11}+c_{12}\right).$$

Substituting Equations (5) and (6) into Equations (12) and (13), we obtain

(15) $$T_{3}={\bar{c}}_{33}S_{3}={\bar{c}}_{33}\left(w_{,3}+x_{2}\psi_{,3}\right),T_{4}=c_{44}S_{4}-e_{15}^{\left(i\right)}E_{2}=c_{44}\left(v_{,3}+\psi \right)+e_{15}^{\left(i\right)}\varphi_{,2},$$

(16) $$D_{3}={\bar{e}}_{33}^{\left(i\right)}S_{3}={\bar{e}}_{33}^{\left(i\right)}\left(w_{,3}+x_{2}\psi_{,3}\right),D_{2}=e_{15}^{\left(i\right)}S_{4}+\varepsilon_{11}E_{2}=e_{15}^{\left(i\right)}\left(v_{,3}+\psi \right)-\varepsilon_{11}\varphi_{,2}.$$

After integrating *T*_3_ over the cross-section, the bending moment *M* is obtained

(17) $$M=\iint x_{2}T_{3}dx_{1}dx_{2}={\bar{c}}_{33}I\psi_{,3}.$$

in which

(18) $$I=\int_{A}x_{2}^{2}dx_{2}=\frac{2}{3}bh^{3}.$$

In general, the derivative of *D*_3_ in the axial direction is much smaller than that of *D*_2_ in the transversal one [44], such that static bending of a piezoelectric semiconductor beam can be partly-decoupled. For bending with shear but not an extension problem, the governing equation can be expressed as [41]

(19) $$Q_{,3}=0$$

in which,

(20) $$Q=M_{,3}={\bar{c}}_{33}I\psi_{,33}.$$

Moreover, Gauss’s law can be written as

(21) $$D_{2,2}=-q\Delta n.$$

## 3. Static Bending Analysis

For a cantilever beam, the boundary conditions are

(22) $$u_{2}\left(0\right)=0,\;\psi \left(0\right)=0;\;Q(L)=F,\;M\left(L\right)=0;\;D_{2}\left(\pm h\right)=0.\;$$

In addition, the following continuity condition at the interface could be expressed as

(23) $$D_{2}^{+}(i)=D_{2}^{-}(i),\;\mathrm{at}\;\mathrm{the}\;i\text{-}\mathrm{th}\;\mathrm{interface}.$$

It is noted that the general solutions of Equations (19)–(21) can be expressed as

(24) $$\psi =-\frac{FL}{{\bar{c}}_{33}I}x_{3}+\frac{F}{2{\bar{c}}_{33}I}x_{3}^{2}.$$

(25) $$v=\frac{FL}{2{\bar{c}}_{33}I}x_{3}^{2}-\frac{F}{6{\bar{c}}_{33}I}x_{3}^{3}+S_{4}x_{3}.$$

(26) $$\Delta n^{\left(i\right)}=C_{2i-1}e^{Bx_{2}}+C_{2i}e^{-Bx_{2}},i=1,2\ldots k,$$

where

(27) $$B={\left(q\frac{\mu_{11}^{n}n_{0}}{\varepsilon_{11}D_{11}^{n}}\right)}^{1/2}$$

Thus, the undefined parameters *C_i_* can be solved with the help of the above boundary and continuity conditions on a computer using MATLAB. Solutions for a bi-layer beam and a three-layer beam are listed below as examples:

(1) for a bi-layer beam,

(28) $$S_{4}=\frac{F}{2bhc_{44}+\frac{4b{\left(e_{15}^{\left(1\right)}\right)}^{2}\left(e^{Bh}-1\right)}{B\varepsilon_{11}\left(e^{Bh}+1\right)}};$$

(29) $$C_{1}=C_{4}=\frac{e_{15}^{\left(1\right)}\mu_{11}^{n}n_{0}S_{4}}{\varepsilon_{11}D_{11}^{n}B\left(e^{Bh}+1\right)},C_{2}=C_{3}=-e^{Bh}C_{1}.$$

(2) for a three-layer beam,

(30) $$S_{4}=\frac{\left(e^{2Bh/3}+1\right)B\varepsilon_{11}F}{3b\left(\left(Bc_{44}h\varepsilon_{11}+2{\left(e_{15}^{\left(1\right)}\right)}^{2}\right)e^{2Bh/3}+Bc_{44}h\varepsilon_{11}-2{\left(e_{15}^{\left(1\right)}\right)}^{2}\right)};$$

(31) $$C_{1}=\frac{e_{15}^{\left(1\right)}\mu_{11}^{n}n_{0}S_{4}e^{-Bh/3}}{\varepsilon_{11}D_{11}^{n}B\left(e^{2Bh/3}+1\right)},C_{2}=-e^{4Bh/3}C_{1},C_{3}=-e^{2Bh/3}C_{1},C_{4}=-C_{3},C_{5}=-C_{2},C_{6}=-C_{1}.$$

## 4. Numerical Results and Discussion

As an example, the number of layers is taken from *k* = 1 to *k* = 3. The related parameters are set as L=3 μm, h=b=0.2 μm, F=−0.8 nN, and $n_{0}=N_{D}=10^{21}\;m^{-3}$, unless specially stated. The other material constants of ZnO are taken from [45]:

(32) $$c_{11}=207\;\mathrm{GPa},\;c_{12}=117.7\;\mathrm{GPa},\;c_{13}=106.1\;\mathrm{GPa},\;c_{33}=209.5\;\mathrm{GPa},\;c_{44}=44.8\;\mathrm{GPa},\;\;e_{15}=-0.45\;C/m^{2},\;e_{31}=-0.51\;C/m^{2},\;e_{33}=1.22\;C/m^{2},\;\varepsilon_{11}=7.77\;\varepsilon_{0},\;\varepsilon_{33}=8.91\;\varepsilon_{0},\;D_{11}^{n}=5.2\times 10^{-4}\;m^{2}/s,\;\mu_{11}^{n}=0.02\;m^{2}/\mathrm{Vs}.$$

**Figure 2:**
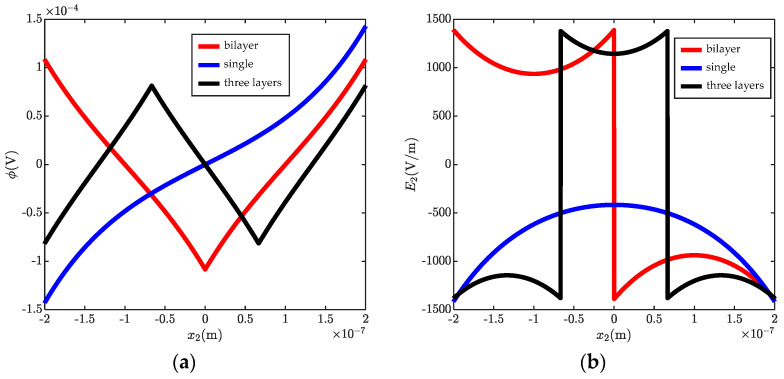

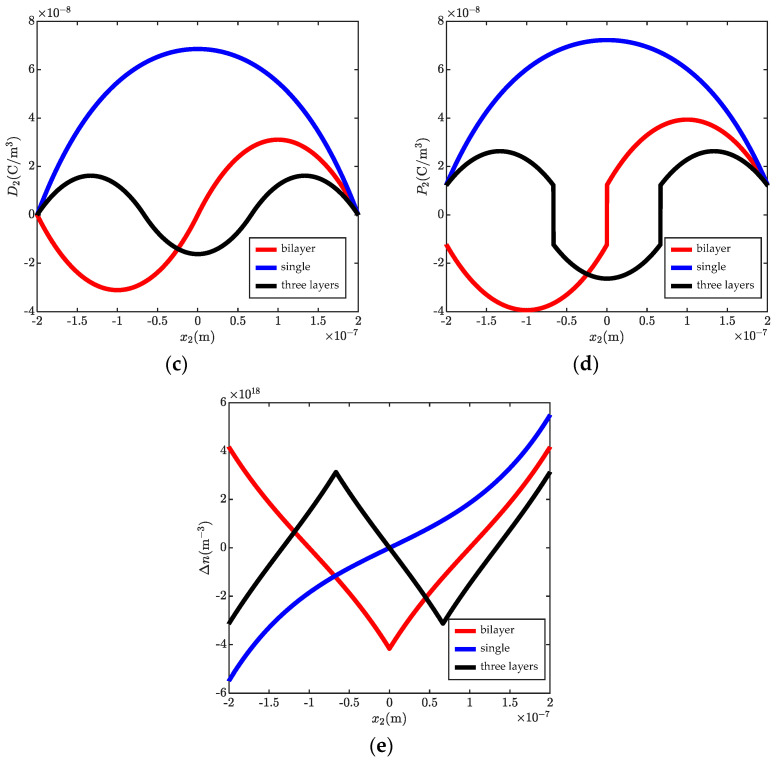
Electrostatics in a cantilever ZnO beam with different numbers of layers. (**a**) electric potential ϕ, (**b**) electric field $E_{2}$, (**c**) electric displacement $D_{2}$, (**d**) polarization $P_{2}$, (**e**) carrier concentrations Δn.

Figure 2 shows the fluctuations of electric potential (ϕ), electric field $\left(E_{2}\right)$, electric displacement $\left(D_{2}\right)$, polarization $\left(P_{2}\right)$, and carrier concentrations (Δn) in the different numbers of layers. It is noted that the point without fluctuation of carrier concentrations is set as a reference point to determine the electric potential. It is observed from Figure 2a that there is no potential barrier or potential well appearing in a single layer ZnO beam. However, a potential well appears in the bi-layer case and a potential barrier accompanying a potential well is showed in a three-layer case. Thus, it is easy to conclude that the potential barrier and potential well will alternate with the increasing number of layers. In addition, it is obvious that the barrier peaks and the well troughs appear just at the interface. This is because of the discontinuous electric polarization there, such that polarized charges (Δρ) are generated at the interface. As shown in Figure 2d, negative polarized charges are generated in a bi-layer beam, which can be calculated from $\Delta \rho (0)=P(0^{-})-P(0^{+})$. The same is true for the three-layer beam; positive polarized charges are generated between the first and second layers, and negative ones appear between the second and third layers. Thus, ϕ decreases near the negative polarized charges and increases near the positive ones. Correspondingly, the electric potential energy of electrons is increased due to the negative polarized charges, which decrease the concentration of carriers (shown in Figure 2e). In contrast, the carrier concentrations are increased with the decreasing electric potential energy of electrons. As reported in Ref. [46], the local stress distribution induces a similar potential barrier and potential well structure, which would forbid the passing of low-energy mobile charges. Therefore, it is found that the maximum fluctuation of carrier concentration decreases with the increasing number of layers. Moreover, typical characteristics in the hetero-structure are shown in the homo-structure with reversed *c*-axial, such as the discontinuity electric field shown in Figure 2b. This phenomenon is similar to the multilayered graphene structure, which could present typical characteristics of the hetero-structures due to the interlaminar Van der Waals forces. However, the interface polarized charges are shown in this paper. From Figure 2b, it is also observed that *E*_2_ is enhanced because the interface polarized charges weaken the shielding effect of carriers. This is consistent with some experimental studies which found that hetero-structures may reduce the phenomenon of electric leakage [47]. In this study, the configuration of a multilayered beam with opposite c-axis directions can be seen as a special hetero-structure. Therefore, the present study provides guidance to developing new piezotronic devices such as nanogenerators, heterojunctions, transistors, etc. Finally, as shown in Figure 2c, it is further observed that *D*_2_ vanishes at the interface, such that the charge neutrality condition is satisfied in each layer.

**Figure 3:**
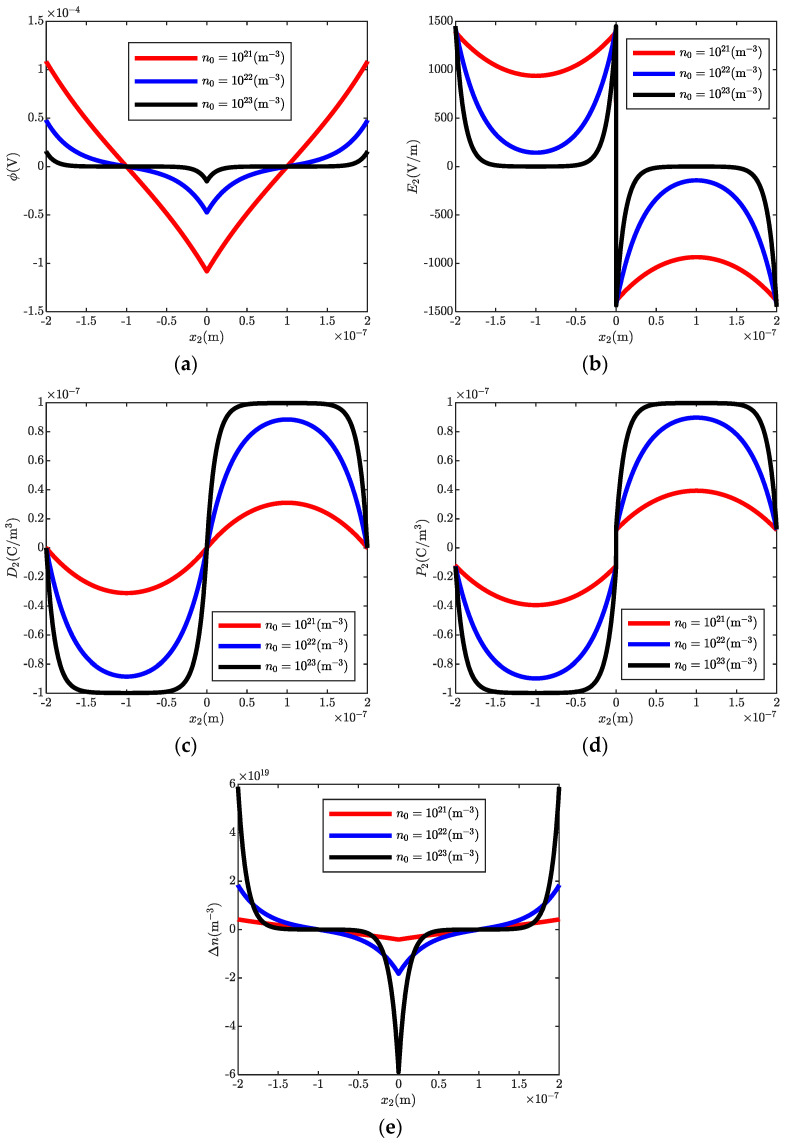
Electrostatics in a ZnO bi-layer with different doping concentrations. (**a**) electric potential ϕ, (**b**) electric field $E_{2}$, (**c**) electric displacement $D_{2}$, (**d**) polarization $P_{2}$, (**e**) carrier concentrations Δn.

Figure 3 shows the electrostatics in a cantilever ZnO bi-layer with different doping concentrations. The carriers are driven to the upper (lower) surface of the first (second) layer due to the effect of the transverse electric field. It is observed that a platform appears at the region (−1.5, −0.5) and (0.5, 1.5) with the increase of initial carrier concentrations. This is because the shielding effect of carriers is enhanced by the increasing doping concentrations, such that the polarized electric field is screened. It is noted that this region is determined by the Debye-Hückel length. However, the Debye-Hückel length is inversely proportional to doping concentrations [43]. Thus, the effective fluctuation region is shortened with increased doping concentrations. In addition, it is obvious that the doping concentrations have a minute effect on the magnitude of the polarized charges at the interface.

**Figure 4:**
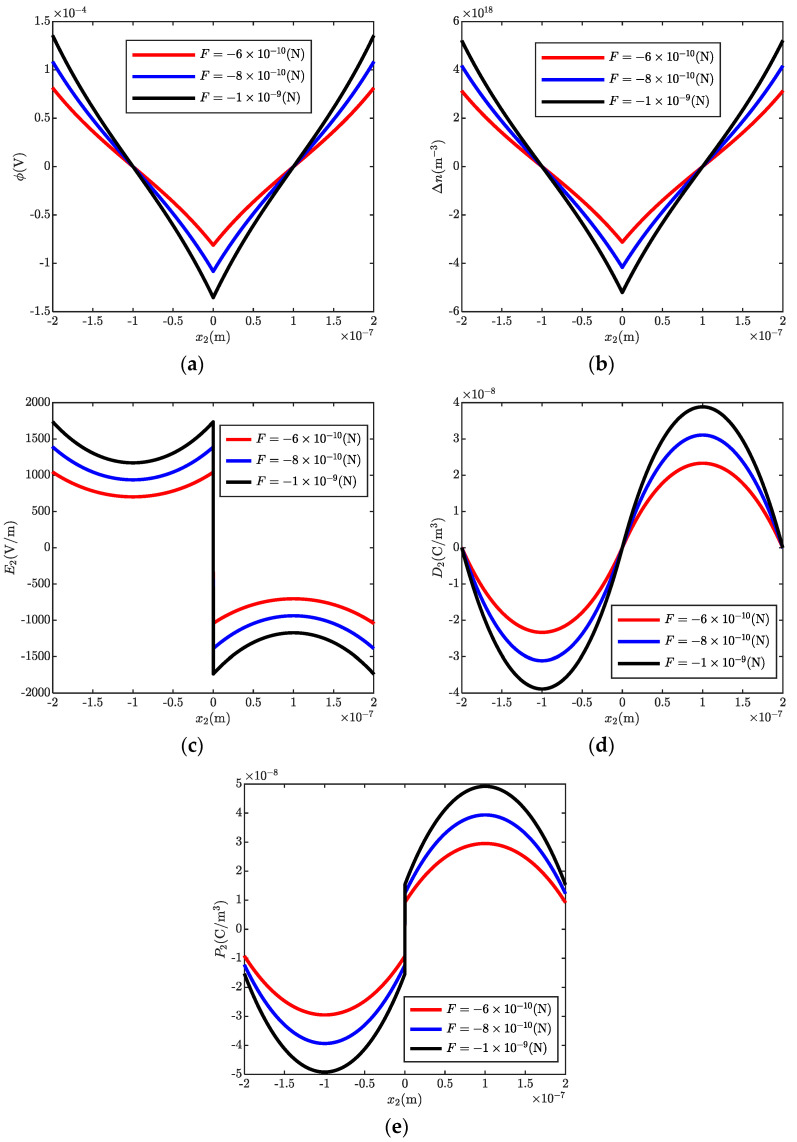
Electrostatics in a ZnO bi-layer with different transverse shear forces. (**a**) electric potential ϕ, (**b**) carrier concentrations Δn, (**c**) electric field $E_{2}$, (**d**) electric displacement $D_{2}$, (**e**) polarization $P_{2}$.

**Figure 5:**
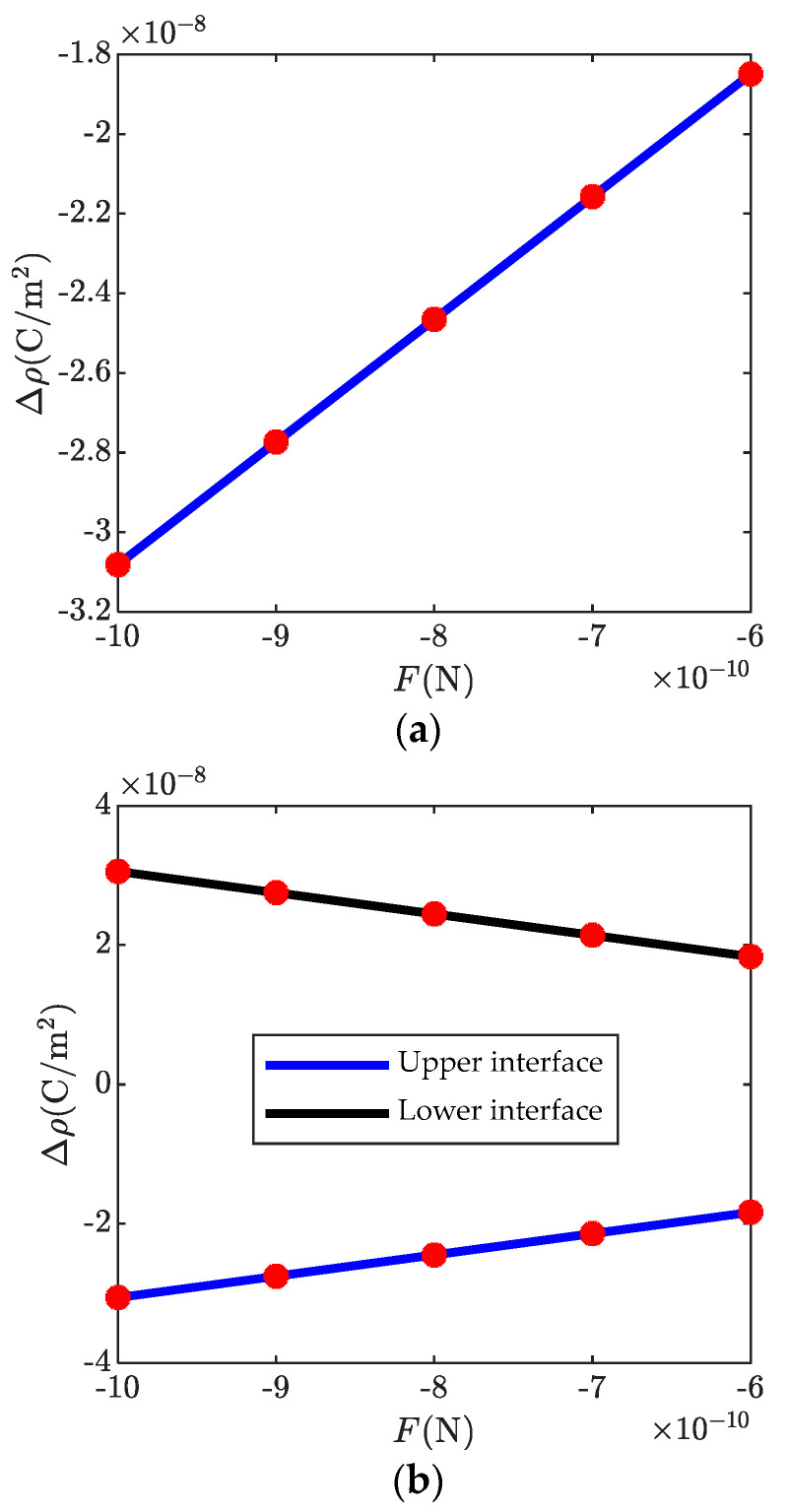
The polarized charges vs. transverse shear forces: (**a**) bi-layer, (**b**) three-layer.

Figure 4 shows ϕ, Δn, $E_{2}$, $D_{2}$, and $P_{2}$ in a ZnO bi-layer with different transverse shear forces. As expected, the magnitudes of all the electrostatics become stronger with the increasing transverse shear forces. Thus, the potential barrier height and the well depth are strengthened with increasing end forces, such that more energy is needed for the passing electrons. This characteristic can be used to design new piezotronic devices. In addition, it differs from Figure 3d because the magnitude of the transverse shear force has an obvious effect on the polarized charges. The amount of the polarized charges vs. the magnitude of the applied end force is shown in Figure 5. It is observed that the amount of the polarized charges is linearly related to the end force.

## 5. Conclusions

A multilayered piezoelectric semiconductor beam model was developed to expose the dramatic interaction between electromechanical fields and carriers in the cross-section. Numerical examples were carried out in a multilayered ZnO cantilever with different numbers of layers and different doping concentrations, and subjected to different end forces. After in-depth study, some observations can be drawn as follows:

(1) The typical characteristic of hetero-structures is shown in the present structure, with alternating opposite *c*-axis along the thickness direction such that polarized charges occur at the interface;
(2) A series of potential barrier/well structures are obtained by the multi-layer beam configuration, which is of significance in tuning and designing the structure performance in terms of number of layers, doping concentrations, and magnitudes of end forces. In addition, these special potential configurations can forbid the passing of low-energy mobile charges;
(3) The induced polarized charges effectively weaken the shielding effect of carrier redistribution. This means the phenomenon of electric leakage is improved;
(4) The amount of the polarized charges is linearly related to the end forces;
(5) Overall, the present study presents guidance for the research and development of new piezotronic devices such as nanogenerators, heterojunctions, transistors, etc.

## References

[1] Tiwari B., Babu T., Choudhary R. (2021). Piezoelectric lead zirconate titanate as an energy material: A review study. Mater. Today Proc..

[2] Sukumaran S., Chatbouri S., Rouxel D., Tisserand E., Thiebaud F., Zineb T. (2021). Recent advances in flexible PVDF based piezoelectric polymer devices for energy harvesting applications. J. Intell. Mater. Syst. Struct..

[3] Mariello M., Qualtieri A., Mele G., Vittorio M. (2021). Metal-Free multilayer hybrid PENG based on soft electrospun/sprayed membranes with cardanol additive for harvesting energy from surgical face masks. ACS Appl. Mater. Interfaces.

[4] Mariello M., Guido F., Algieri L., Mastronardi M., Qualtieri A., Pisanello F., Vittorio M. (2021). Microstructure and electrical properties of novel piezo-optrodes based on thin-film piezoelectric aluminium nitride for sensing. IEEE Trans. Nanotechnol..

[5] Wang Z. (2012). Piezotronics and Piezo-Phototronics.

[6] Zhu G., Yang R., Wang S., Wang Z. (2010). Flexible high-output nanogenerator based on lateral ZnO nanowire array. Nano Lett..

[7] Wang L., Wang Z. (2021). Advances in piezotronic transistors and piezotronics. Nano Today.

[8] Gao Y., Wang Z. (2007). Electrostatic potential in a bent piezoelectric nanowire. The fundamental theory of nanogenerator and nanopiezotrionics. Nano Lett..

[9] Falconi C. (2019). Piezoelectric nanotransducers. Nano Energy.

[10] Zhang Y., Liu Y., Wang Z. (2011). Fundamental theory of piezotronics. Adv. Mater..

[11] Peng Y., Que M., Lee H., Bao R., Wang X., Lu J., Yuan Z., Li X., Tao J., Sun J. (2019). Achieving high-resolution pressure mapping via flexible GaN/ZnO nanowire LEDs array by piezo-phototronic effect. Nano Energy.

[12] Jiang C., Jing L., Huang X., Liu M., Du C., Liu T., Pu X., Hu W., Wang Z. (2017). Enhanced solar cell conversion efficiency of InGaN/GaN multiple quantum wells by piezo-phototronic effect. ACS Nano.

[13] Zhang Y., Yang Y., Wang Z. (2012). Piezo-phototronics effect on nano/microwire solar cells. Energy Environ. Sci..

[14] Wu W., Wang Z. (2011). Piezotronic nanowire-based resistive switches as programmable electromechanical memories. Nano Lett..

[15] Wang Z. (2003). Nanobelts, nanowires, and nanodiskettes of semiconducting oxides-from materials to nanodevices. Adv. Mater..

[16] Wang Z. (2010). Piezopotential gated nanowire devices: Piezotronics and piezo-phototronics. Nano Today.

[17] Kumar B., Kim S. (2011). Recent advances in power generation through piezoelectric nanogenerators. J. Mater. Chem..

[18] Fang K., Li P., Qian Z. (2021). Static and dynamic analysis of a piezoelectric semiconductor cantilever under consideration of flexoelectricity and strain gradient elasticity. Acta Mech. Solida Sin..

[19] Qu Y., Jin F., Yang J. (2022). Bending of a flexoelectric semiconductor plate. Acta Mech. Solida Sin..

[20] Sharma J., Sharma K., Kumar A. (2010). Surface waves in a piezoelectric-semiconductor composite structure. Int. J. Solids Struct..

[21] Othmani C., Takali F., Njeh A. (2017). Modeling of phase velocity and frequency spectrum of guided Lamb waves in piezoelectric-semiconductor multilayered structures made of AlAs and GaAs. Superlattices Microstruct..

[22] Othmani C., Takali F., Njeh A., Ghozlen M. (2016). Study of the influence of semiconductor material parameters on acoustic wave propagation modes in GaSb/AlSb bi-layered structures by Legendre polynomial method. Phys. B Condens. Matter.

[23] Luo Y., Zhang C., Chen W., Yang J. (2018). Piezopotential in a bended composite fiber made of a semiconductive core and of two piezoelectric layers with opposite polarities. Nano Energy.

[24] Cheng R., Zhang C., Chen W., Yang J. (2018). Piezotronic effects in the extension of a composite fiber of piezoelectric dielectrics and nonpiezoelectric semiconductors. J. Appl. Phys..

[25] Jiao F., Wei P., Zhou Y., Zhou X. (2019). Wave propagation through a piezoelectric semiconductor slab sandwiched by two piezoelectric half-spaces. Eur. J. Mech. A/Solids.

[26] Tian R., Liu J., Pan E., Wang Y. (2020). SH waves in multilayered piezoelectric semiconductor plates with imperfect interfaces. Eur. J. Mech. A/Solids.

[27] Tian R., Nie G., Liu J., Pan E., Wang Y. (2021). On Rayleigh waves in a piezoelectric semiconductor thin film over an elastic half-space. Int. J. Mech. Sci..

[28] Liang C., Zhang C., Chen W., Yang J. (2020). Effects of magnetic fields on PN junctions in piezomagnetic–piezoelectric semiconductor composite fibers. Int. J. Appl. Mech..

[29] Qu Y., Jin F., Yang J. (2020). Effects of mechanical fields on mobile charges in a composite beam of flexoelectric dielectrics and semiconductors. J. Appl. Phys..

[30] Nafday D., Saha T. (2013). Magnetism of an adatom on bilayer graphene and its control: A first-principles perspective. Phys. Rev. B.

[31] Mccann E., Koshino M. (2013). The electronic properties of bilayer graphene. Rep. Prog. Phys..

[32] Cheng R., Zhang C., Chen W., Yang J. (2020). Temperature effects on PN junctions in piezoelectric semiconductor fibers with thermoelastic and pyroelectric couplings. J. Electron. Mater..

[33] Ren C., Wang K., Wang B. (2021). Analysis of piezoelectric PN homojunction and heterojunction considering flexoelectric effect and strain gradient. J. Phys. D Appl. Phys..

[34] Yang G., Zhang G., Zhou H., Qi Z. (2009). Synchrotron radiation assistant MOCVD deposition of ZnO films on Si substrate. Appl. Surf. Sci..

[35] Liang Y., Hu Y. (2020). Effect of interaction among the three time scales on the propagation characteristics of coupled waves in a piezoelectric semiconductor rod. Nano Energy.

[36] Dokmeci M. (1974). A theory of high frequency vibrations of piezoelectric crystal bars. Int. J. Solids Struct..

[37] Mindlin R. (1976). Low frequency vibrations of elastic bars. Int. J. Solids Struct..

[38] Chou C., Yang J., Hwang Y., Yang H. (1991). Analysis on vibrating piezoelectric beam gyroscope. Int. J. Appl. Electromagn. Mater..

[39] Yang J. (1998). Equations for the extension and flexure of a piezoelectric beam with rectangular cross section and applications. Int. J. Appl. Electromagn. Mater..

[40] Yang W., Hu Y., Pan E. (2019). Tuning electronic energy band in a piezoelectric semiconductor rod via mechanical loading. Nano Energy.

[41] Zhang C., Wang X., Chen W., Yang J. (2018). Bending of a cantilever piezoelectric semiconductor fiber under an end force. Generalized Models and Non-classical Approaches in Complex Materials 2.

[42] Yang J., Zhou H. (2005). Amplification of acoustic waves in piezoelectric semiconductor plates. Int. J. Sol. Struct..

[43] Sze S., Kwok K. (2007). Physics of Semiconductor Devices.

[44] Fan S., Liang Y., Xie J., Hu Y. (2017). Exact solutions to the electromechanical quantities inside a statically-bent circular ZnO nanowire by taking into account both the piezoelectric property and the semiconducting performance: Part I—Linearized analysis. Nano Energy.

[45] Liang Y., Fan S., Chen X., Hu Y. (2018). Nonlinear effect of carrier drift on the performance of an n-type ZnO nanowire nanogenerator by coupling piezoelectric effect and semiconduction. Beilstein J. Nanotechnol..

[46] Fan S., Hu Y., Yang J. (2019). Stress-induced potential barriers and charge distributions in a piezoelectric semiconductor nanofiber. Appl. Math. Mech..

[47] Lee K., Bae J., Kim S., Lee J., Yoon G., Gupta M., Kim S., Kim H., Park J., Kim S. (2014). Depletion width engineering via surface modification for high performance semiconducting piezoelectric nanogenerators. Nano Energy.

